# Parasite associations predict infection risk: incorporating co-infections in predictive models for neglected tropical diseases

**DOI:** 10.1186/s13071-020-04016-2

**Published:** 2020-03-16

**Authors:** Nicholas J. Clark, Kei Owada, Eugene Ruberanziza, Giuseppina Ortu, Irenee Umulisa, Ursin Bayisenge, Jean Bosco Mbonigaba, Jean Bosco Mucaca, Warren Lancaster, Alan Fenwick, Ricardo J. Soares Magalhães, Aimable Mbituyumuremyi

**Affiliations:** 1 UQ Spatial Epidemiology Laboratory, School of Veterinary Science, The University of Queensland, Gatton, QLD 4343 Australia; 2 Children Health and Environment Program, Child Health Research Centre, The University of Queensland, South Brisbane, QLD 4101 Australia; 3 Neglected Tropical Diseases and Other Parasitic Diseases Unit, Malaria and Other Parasitic Diseases Division, Rwanda Biomedical Center, Kigali, Rwanda; 4 Schistosomiasis Control Initiative (SCI), Department of Infectious Diseases Epidemiology, Imperial College, London, UK; 5 Microbiology Unit, National Reference Laboratory (NRL) Division, Rwanda Biomedical Center, Ministry of Health, Kigali, Rwanda; 6 The END Fund, 2 Park Avenue, 18th Floor, New York, NY 10016 USA; 7 Malaria and Other Parasitic Diseases Division, Rwanda Biomedical Center, Ministry of Health, Kigali, Rwanda

**Keywords:** Conditional random fields, Neglected tropical disease, Parasite co-infection, *Schistosoma mansoni*, Soil-transmitted helminths, Spatial epidemiology

## Abstract

**Background:**

Schistosomiasis and infection by soil-transmitted helminths are some of the world’s most prevalent neglected tropical diseases. Infection by more than one parasite (co-infection) is common and can contribute to clinical morbidity in children. Geostatistical analyses of parasite infection data are key for developing mass drug administration strategies, yet most methods ignore co-infections when estimating risk. Infection status for multiple parasites can act as a useful proxy for data-poor individual-level or environmental risk factors while avoiding regression dilution bias. Conditional random fields (CRF) is a multivariate graphical network method that opens new doors in parasite risk mapping by (i) predicting co-infections with high accuracy; (ii) isolating associations among parasites; and (iii) quantifying how these associations change across landscapes.

**Methods:**

We built a spatial CRF to estimate infection risks for *Ascaris lumbricoides*, *Trichuris trichiura*, hookworms (*Ancylostoma duodenale* and *Necator americanus*) and *Schistosoma mansoni* using data from a national survey of Rwandan schoolchildren. We used an ensemble learning approach to generate spatial predictions by simulating from the CRF’s posterior distribution with a multivariate boosted regression tree that captured non-linear relationships between predictors and covariance in infection risks. This CRF ensemble was compared against single parasite gradient boosted machines to assess each model’s performance and prediction uncertainty.

**Results:**

Parasite co-infections were common, with 19.57% of children infected with at least two parasites. The CRF ensemble achieved higher predictive power than single-parasite models by improving estimates of co-infection prevalence at the individual level and classifying schools into World Health Organization treatment categories with greater accuracy. The CRF uncovered important environmental and demographic predictors of parasite infection probabilities. Yet even after capturing demographic and environmental risk factors, the presences or absences of other parasites were strong predictors of individual-level infection risk. Spatial predictions delineated high-risk regions in need of anthelminthic treatment interventions, including areas with higher than expected co-infection prevalence.

**Conclusions:**

Monitoring studies routinely screen for multiple parasites, yet statistical models generally ignore this multivariate data when assessing risk factors and designing treatment guidelines. Multivariate approaches can be instrumental in the global effort to reduce and eventually eliminate neglected helminth infections in developing countries.
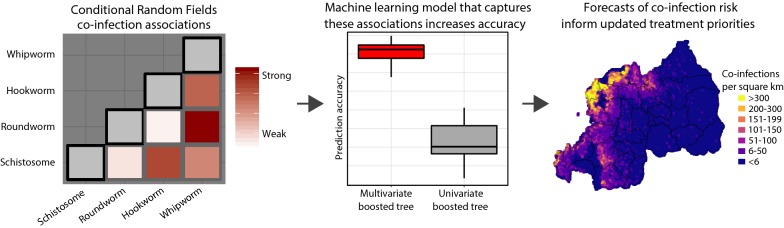

## Background

Helminth parasites cause some of the world’s most important neglected tropical diseases [1–3]. Morbidity attributed to soil-transmitted helminths (STHs; *Ascaris lumbricoides*, *Trichuris trichiura*, *Ancylostoma duodenale* and *Necator americanus*) and trematodes (genus *Schistosoma*) is widespread in impoverished areas with limited access to clean water and sanitation [4, 5]. Some of the world’s highest helminth burdens occur in Africa [6], where up to 300,000 people die from schistosomiasis each year [7] and up to two in three children are infected with an STH [8, 9]. Concomitant infection with multiple parasites (co-infection) is ubiquitous, with parasites often overlapping in distributions and sharing risk factors [10, 11]. Co-infections have important public health implications, as infection by multiple parasites can increase the progression and magnify the severity of parasite-related disease [12, 13].

The pharmaceutical industry, the World Health Organization (WHO) and the scientific community all recognise reduction of helminth-related morbidity as a global priority and work to identify regions in need of anthelmintics [14]. Their goal is to deliver mass drug administration (MDA), which alleviates helminth-related health burdens by reducing prevalence and associated morbidity [15, 16]. Geostatistical models are key for designing MDA programmes by providing decision-support tools to aid delivery to areas of high prevalence [10, 17]. These models are also crucial for deciding between programmes geared towards either sustained control or parasite elimination [18].

Data used to build geostatistical models often comes from national surveys of infections for multiple helminth species deployed over a number of years. Such an approach was recently conducted in Rwanda, a densely populated agriculture-based country in sub-Saharan Africa exhibiting some of the world’s highest helminth burdens [6, 19]. While these surveys often gather information on multiple parasites, identification of high-risk areas typically relies on single-species models [20, 21]. This limits capacity to delineate communities at risk of morbidity due to co-infections and hampers multi-parasite treatment regimens.

The health impacts of parasite co-infections are widely recognised. But while studies have begun accounting for multiple parasites in models, such investigations are few and are limited to either classifying infections into multinomial categories [11, 12, 22, 23] or aggregating total numbers of detected parasite species as counts in a Poisson regression [24]. Unfortunately, neither of these approaches provides insights into possible associations among parasite infection risks. Effects of each parasite’s presence on one another’s infection risk are not measured or are not comparable to other predictors, meaning important questions such as “Does infection with one parasite decrease risk of infection with another?” and “Do parasite associations have predictive utility for forecasting geographical disease risk?” are not fully explored (but see [25]). In ecology, the study of how and when species co-occur has long been recognised as important to our understanding of biodiversity generation, predator-prey interactions and community assembly [26–28]. So-called ‘Joint Species Distribution Models’ (JSDMs) have become a popular class of tools to gain insights into species co-occurrences by moving beyond single-species correlative distribution models to explicitly capture covariance relationships among species using multivariate residual matrices [27, 29]. While most parasite epidemiology studies still focus on detecting environmental or demographic correlates of infection risk for individual parasite species, studies that use JSDMs to draw biological conclusions about relationships among parasite infection probabilities are becoming increasingly relevant to our understanding of multi-parasite epidemiology [30–32]. Yet despite the advanced insights that can be gained from JSDMs, a common drawback is that they generally lack capacity to explore how parasite associations change across scales. Both the strength and direction of interspecific associations can vary along environmental gradients [33]. This is a problem that is encountered in many fields of multivariate analysis. For example, the increasingly-important task of accurately quantifying levels of landscape change using high-resolution satellite images is reliant on the underlying model’s ability to capture how interactions among neighbouring image pixels change over time [34].

Addressing these issues requires multivariate approaches that (i) isolate associations among co-occurring variables and (ii) estimate how associations change by allowing them to be conditional upon external covariates [33]. A type of machine learning graphical network model known as conditional random fields (CRFs) is an approach that allows for such inference [35, 36]. CRFs incorporate the type of multivariate data that is routinely gathered using diagnostic tests but is commonly ignored when modelling risk. Rather than aggregating infections, CRFs parameterize interactions between network nodes to gain insights into predictors of each node’s occurrence. CRFs can also identify how co-infection risks might change across landscapes, an attractive property that could improve our understanding of how infection risks are locally shaped and ultimately lead to better management strategies. Translated to helminths, CRFs can answer questions such as “What is the risk that an individual will carry a particular parasite, and how is this risk related to both environmental conditions and to the presence of other parasites?” By applying CRFs to infection data in Rwandan schoolchildren, we showcase how these models are powerful tools for predictive analyses and for identification of populations in need of intervention.

## Methods

### A national survey of helminth parasite infections in Rwanda

We used data from a survey designed to detect helminth infection prevalence in Rwandan schoolchildren. A national prevalence mapping effort was designed by considering groups of administrative mapping units [37]. Schools were chosen to ensure sample sizes in each unit were representative of the population sizes in their respective geographical areas. The survey was administered in June–July 2014 across 186 schools. For each child, age and sex were recorded and the presence-absence of helminth infections assessed by examination of duplicate 41.7 mg smears prepared from a single stool using the Kato-Katz (KK) method [38]. Faecal egg counts were estimated to calculate eggs per gram of faeces (epg). KK smears were assessed by two technicians, with infection denoted as positive if at least one egg was detected. Two experts randomly re-assessed 10% of smears for quality control.

### Environmental variables

Helminth parasite distributions can be influenced by both climatic and environmental heterogeneity [39, 40]. Detecting environmental correlates is an important step for developing interventions aimed at reducing burdens [41–43]. We extracted remote-sensed measurements for six variables that reflect variation in temperature, vegetation, moisture and the presence of water bodies, all of which can impact survival of STH larvae [44] or distributions of molluscs that act as intermediate hosts for *Schistosoma* parasites [45]. These were: land surface temperature (LST); the normalized difference vegetation index (NDVI); the normalized difference water index (NDWI); elevation; surface soil moisture; and the proportion of cells classified as cropland (variable sources and spatial resolutions are shown in Table 1). Time-variant variables (LST, NDVI, NDWI and soil moisture) were calculated as mean post-rainy season variables.

**Table 1:** Environmental and demographic predictor variables used in analysis

Predictor variable	Analysis resolution	Source
Net difference vegetation index (NDVI)	10 km	MODIS
Net difference water index (NDWI)	10 km	MODIS
Land surface temperature (LST)	10 km	MODIS
Elevation	10 km	Amazon web services
Surface soil moisture	10 km	NASA SMAP
Proportion of landcover classified as cropland (Categories 11 and 12)	10 km	MODIS
Human population density	10 km	Facebook Connectivity Laboratory and CIESIN
Proportion of human settlements classified as rural (Category 1)	10 km	Global human settlement layer
Proportion of households with electricity	District	DHS Malaria Indicator Survey
Proportion of households with access to flushable toilet	District	DHS Malaria Indicator Survey
Proportion of households with access to clean drinking water	District	DHS Malaria Indicator Survey

*Notes*: NDVI, NDWI, LST and surface soil moisture were calculated as post-rainy season averages (December to February). Other predictors included in analysis were the child’s age and sex

### Demographic variables

Differences in access to and use of sanitation can also lead to wide variability in environmental contamination by infective parasite stages [15]. Indices of poverty and sanitation/hygiene are important for modelling helminth infection risk [46, 47]. We used household questionnaire data from the Demographic and Health Survey (DHS) Programme’s 2017 Rwandan Malaria Indicator Survey [48] to calculate three district-level variables capturing variation in poverty/sanitation access. These were the proportion of households in the district reported to have: electricity; access to a flushable toilet; and access to clean drinking water. We included two variables reflecting human population sizes to adjust for differences in environmental contamination that may occur in areas with dense populations of at-risk people. These were: human population density and the proportion of cells classified as ‘rural’ settlements (Table 1). All predictors apart from DHS variables were calculated as 10 km averages around school centroids to maintain consistent coverage and allow for the fact that some children may travel to school.

### Conditional random fields to model infection risk

Undirected graphical networks offer flexible tools for estimating direct and indirect associations between co-occurring organisms and for disentangling influences of interspecific associations and environment on species’ distributions. We used one such approach to assess whether inclusion of co-infection information can aid managers in the design of treatment regimes. Markov random fields (MRFs) are an important subgroup of statistical network models called probabilistic graphical models [49]. The term ‘graphical’ refers to a property of these models whereby they can effectively represent complex distributions as compact network graphs that consist of two primary types of elements: nodes, which correspond to the observed variables within the data; and the edges between the nodes, which correspond to the probabilistic interactions between variables that need to be estimated. In MRFs, these interaction edges are considered undirected, meaning that the effect of one node on another is reciprocal. The absence of an edge between two nodes in the estimated graph indicates that the two variables are conditionally independent of one another, while the presence of an edge indicates that the two connected nodes are conditionally dependent after accounting for possible effects of all other nodes in the graph [36, 50]. In binary MRFs, as is this case with our parasite presence-absence data, these conditional dependencies are typically estimated using a joint probability function that defines the relative probability of observing a given vector of node presences (1s) and absences (0s) conditional on the presences and absences of all other nodes. A crucial property of the CRF, which is an extension of MRFs, is that it allows these dependencies among node variables to be conditional on external covariates, where values for the edges that connect nodes in the graph can change in the presence of covariates [33, 36].

A major challenge of maximizing the joint likelihood in a CRF is that this requires the simultaneous estimation of a large number of coefficients in addition to a complex normalizing constant that grows exponentially with the number of nodes in the graph [36]. Using separate models to maximize the conditional likelihood of each node given the remaining nodes and covariates, rather than attempting to maximize the joint likelihood, is a lucrative approximation option that has been widely adopted throughout the statistical literature [36, 51]. For binary CRFs, a number of statistical investigations have shown that an appropriate node-wise approximation is to formulate the graph as a collection of separate logistic regressions [36, 50]. This is advantageous as multivariable logistic regressions yield conditional coefficients that closely relate to a fully specified CRF and can easily be estimated using widely available software and computational resources. In each regression, the binary node of interest is the outcome variable and all other variables in the graph, along with all covariates, are included as predictor variables. Once each nodewise regression has been fit, approximation of the graph can be achieved by symmetrising the corresponding coefficients (i.e. the effect of parasite 1 on parasite 2 must be the same as the effect of parasite 2 on parasite 1). If the number of parameters in each separate regression is large, it is often useful to employ variable selection routines that can induce sparsity by penalizing some of the coefficients toward zero [50].

In our CRF model, we used as input data for the nodes a matrix of individual-level parasite presence-absence vectors (1s and 0s for each parasite in each child that was surveyed). The model design matrix for each separate regression was constructed by cross-multiplying all combinations of co-occurring parasites (binary vectors) and covariates, meaning that each parasite-specific regression included terms for the presence-absence of other parasites, all covariates, and interaction effects among other parasites and the covariates. In other words, if our model contained two parasites and one covariate, the regression for parasite 1 would estimate coefficients for the effects of ‘parasite 2’, the ‘covariate’ and the ‘parasite 2 × covariate’ interaction. For the covariates, we included a matrix that captured values for the environmental and demographic covariates listed above. Because cross-multiplication for all of these covariates resulted in a large number of coefficients, estimating all of these coefficients in a typical logistic regression would lead to problems of overfitting. Parameterization of each parasite’s likelihood was therefore estimated using a regularized logistic regression that employed the Least Absolute Shrinkage and Selection Operator (LASSO) to force coefficients to zero if their effects were minimal. This was done *via* a supervised machine-learning procedure that used 10-fold cross-validation to minimise cross-validated error [52]. Coefficients representing conditional dependence for each pair of parasites, and coefficients representing effects of covariates on this dependence, were symmetrised by taking means of the corresponding estimates. All coefficients can be interpreted as effects on a parasite’s log odds, exactly as they would be from a standard logistic regression.

A total of 9251 faecal samples were taken across 186 schools. However, a number of observations were missing age and/or infection information for at least one parasite. These were excluded, resulting in parasite binary presence/absence data for 8786 children sampled across 177 schools (4387 boys; 4399 girls), with the number of children per school ranging from 43 to 50. Ages ranged 8 to 18 years (mean = 13.35; standard deviation = 0.77). Initial predictors tested for inclusion were: age, sex and the environmental/demographic geographical variables listed in Table 1. Continuous predictors were standardized to unit variance. Multicollinearity was accounted for by eliminating the most highly correlated continuous predictor (i.e. the predictor that showed a higher number of strong correlations) from those pairs whose Pearsonʼs correlations were > 0.7 following recommendations by Dormann et al. [53] that correlations above this value can severely distort model estimation and resulting prediction. Two variables, NDWI and LST, were removed during this process. Because spatial autocorrelation is a common feature of helminth risk [11, 17, 21] and has been demonstrated for STH risk in Rwanda [37], we accounted for spatial effects by including Gaussian process spatial regression splines in the linear predictor of each regularized regression.

This cyclic CRF approach is a strongly justified tool for investigating co-infections as it relies on the same underlying assumptions of common logistic regressions but can tackle the crucial, neglected aim of detecting whether any pair of parasites is associated *after* accounting for each parasite’s associations with all other parasites. In addition, CRFs allow one to explore these coefficients further by asking whether these associations among parasites change as covariates increase or decrease. Importantly, datasets such as the one we used here are commonly generated by many multi-pathogen surveillance and multi-species monitoring programmes, and so our method can be easily applied to a variety of contexts. Indeed, other critical examples of our methodology have already demonstrated that viral co-infections provide insights into the timing of Hendra virus emergence events [54] and that the compositions of avian blood parasite communities, marshland arthropod communities and wildlife microbiome profiles are the products of complex factors including biotic associations and environmental variation [33, 55]. However, a drawback of the CRF approach is that prediction of infection risk for unsampled areas is challenging due to the requirement that at least some parasite presence-absence information needs to be available for the unsampled area. The machine learning literature offers many examples of ‘ensemble’ learning methods that combine predictions from multiple supervised algorithms to take advantage of the fact that many different data-adaptive computational methods perform differently under different data-generating scenarios [56–58].

In this study, prediction for unsampled areas was accomplished using one such ensemble approach. We simulated from the CRF’s posterior distribution with a multivariate boosted regression tree that was designed to maximise the importance of predictors that influence covariance in outcomes [59], which were parasite infection risks in our case. Training a multivariate boosted regression tree using environmental and demographic features as covariates and CRF probability predictions as outcomes capitalised on the CRF’s primary purpose of detecting conditional infection risks as well as the regression tree’s primary purpose of learning complex, non-linear covariate interactions to generate accurate spatial risk predictions for unsampled areas [60]. Learning parameters were: maximum trees = 1000, learning rate = 0.01 and interaction depth = 3. CRF fits and diagnostics were performed using functions in the open-source *MRFcov* R package [61].

### Assessing model fit and conducting a sensitivity analysis

Our CRF ensemble was compared against single parasite boosted regression trees, also known as gradient boosted machines (GBMs), as part of an in-depth sensitivity analysis to assess performance, quantify prediction uncertainty and assess at which stage of the methodology chain the highest uncertainties occurred. GBMs have been used in a range of disease mapping studies and are growing in popularity due to their ability to capture nonlinear associations and higher-order interactions in computationally-efficient, user-friendly algorithms [62, 63]. GBMs used the same environmental and demographic predictors as the CRF ensemble and were tuned using identical learning parameters, ensuring the only major difference between approaches was the absence of additional parasite co-infection information in the GBM. We used cross-validation to assess model fit and quantify prediction uncertainty. Models were trained on the same random subset of 80% of children, with resulting equations used to predict infections for the remaining 20% (5-fold cross-validation). We tested the sensitivity of our results to the choice of data subset by repeating this cross-validation 20 times. This process meant that each model was fit to 100 different subsets of the original data, allowing us to adequately account for variation arising from the fold selection procedure and to calculate robust prediction intervals for presenting uncertainty in spatial risk maps.

We scrutinised each model’s ability to capture information relevant to managers tasked with outlining policies to reduce parasite infection burdens and progress towards elimination. In addition to estimating out-of-sample prediction accuracy, we estimated school-wide area under the curve of the receiver operating characteristics (AUCs) using two probability cut-offs that directly matched to WHO treatment categories (for details of guidelines, see https://www.who.int/news-room/fact-sheets/detail/soil-transmitted-helminth-infections and page 35 in [14]). These cut-offs were 0.50 to denote whether schools that would be considered ‘high-risk’ under WHO guidelines (i.e. schools requiring bi-annual treatment) were identified by the models, and 0.20 to represent schools that would be in need of annual treatment according to WHO. AUCs from 0.70–0.89 indicate reasonable discriminatory power, while values > 0.90 reflect good power. Because *Schistosoma* and *Ancylostoma* parasites were rare, we only calculated AUCs for *A. lumbricoides* and *T. trichiura*. We also calculated an *A. lumbricoides* + *T. trichiura* standardised co-infection ratio (SCR) for each school by dividing the expected number of co-infections (based on the mean national rate) by the observed number. To summarize, translational performances were judged by assessing each model’s (i) out-of-sample classification accuracy for individual children; (ii) ability to discriminate schools into the WHO treatment guideline categories for *A. lumbricoides* and *T. trichiura* (using 50 and 20% prevalence thresholds); and (iii) ability to classify *A. lumbricoides* + *T. trichiura* co-infections and rank schools based on their relative co-infection frequencies.

### Producing smoothed prevalence maps

To produce infection prevalence maps, we divided Rwanda into a spatial grid representing 80,258 cells. Interpolated surfaces for each of our point-based predictors were smoothed onto this grid using generalized additive models that included Gaussian process spatial regression splines. Interpolated surfaces were used for producing spatially smoothed predictions. We constructed 100 conditional prevalence maps for each parasite using the cross-validation procedure specified in *Assessing model fit and sensitivity* to present uncertainties in prevalence projections. For projecting burden of *A. lumbricoides* + *T. trichiura* co-infections in Rwandan schoolchildren, we multiplied the estimated probability of co-infection in each cell by the estimated population size in each cell using the gridded human density raster. These values were multiplied by the proportion of people estimated to be school age (37%; [64]).

### Exploring effects of parasite burdens on risk of co-occurring parasites

We identified a number of positive parasite associations, raising questions about possible underlying biological mechanisms. One question of interest is whether increased within-host burden with one parasite correlates with increased probability of co-infection, either by leaving a child more susceptible to acquiring infections or by stimulating parasite egg production [25]. We addressed this by fitting additional models. For each parasite, we generated separate datasets by filtering observations to only include children infected with that parasite. We then fitted a spatial CRF that did not include this parasite as a binary outcome, but instead included its epg as a predictor. We asked whether epg of focal parasites correlated with changing infection risks for other parasites or with changing parasite associations. As above, 100 models were fit to random subsets containing 80% of observations to capture uncertainty. For CRF models, predictors were considered significant if the 95% credible interval (CI) of their estimated coefficients did not include zero. *P*-values are only reported for the results of Pearsonʼs correlations. All data and R code used to fit CRF and GBM models are included in Additional file 1.

## Results

### CRFs improve prediction of infection and co-infection

We fit CRFs to infection data for four parasites (*Ascaris lumbricoides*, *Trichuris trichiura*, hookworms (*Ancylostoma duodenale* and *Necator americanus*) and *Schistosoma mansoni*) from 8786 children sampled across 177 schools (Additional file 2: Figure S1). Prevalences were as follows: *A. lumbricoides*, 37.54%; *T. trichiura*, 23.16%; hookworm, 4.54%; and *S. mansoni*, 2.07% (Additional file 2: Figure S2). A total of 4212 children were infected with at least one parasite (47.02%). Co-infections were common, with 1753 children infected with at least two parasites (19.57%) and 123 harbouring at least three parasites (1.37%). The spatial CRF showed good fit to the observed data. Across all 100 cross-validation runs, binary predictions were correct for 89–91% of the 35,144 total observations (four parasites across 8786 children) and positive predictive values (the proportion of positive infections that were correctly predicted as positive) ranged from 0.71–0.80.

For each parasite, classification accuracy at the individual child level was similar for the CRF ensemble and single-parasite GBMs (Additional file 2: Figure S3). Both models achieved higher accuracy for predicting the rarer parasites (*Schistosoma* and hookworm; accuracies ranging 0.95–0.98), while accuracies for *A. lumbricoides* and *T. trichiura* were lower but still reflective of good performance (accuracies ranging 0.79–0.87 for these parasites; Additional file 2: Figure S3). When tasked with identifying school-wide prevalence to inform WHO treatment guidelines, the CRF ensemble was superior. For *A. lumbricoides*, AUCs predicted by the CRF and GBMs were similar at a 0.50 cut-off, with both models achieving good discriminatory power (AUCs: 0.90–0.97), but the CRF outperformed the GBM at a 0.20 cut-off (Additional file 2: Figure S4). For *T. trichiura*, the CRF outperformed the GBM at both prevalence thresholds, achieving good discriminatory power at 0.50 and reasonable power at 0.20 (Additional file 2: Figure S4).

The CRF ensemble was also better at predicting *A. lumbricoides* + *T. trichiura* co-infections. At the individual level, the CRF correctly predicted 60–65% of co-infections, compared to 46–54% for GBMs (Additional file 2: Figure S5). This pattern held at the school level, with Pearsonʼs correlations between predicted and observed SCRs showing a stronger positive relationship for the CRF (Correlation *r*_(177)_ = 0.85, 95% CI: 0.84–0.87, *P* < 0.0001) compared to the GBM (Correlation *r*_(177)_ = 0.82, 95% CI: 0.81–0.83, *P* < 0.0001; Additional file 2: Figure S6). Nevertheless, both models missed some high-burden schools, although the CRF fared better in this regard. In total, 46 schools had substantially increased frequencies of co-infections compared to the average (harbouring 1.9–5.6 times the background co-infection prevalence and accounting for the top 25% of observed SCRs). The CRF consistently identified 31 (67%) of these as high-risk, compared to 24 (53%) for the GBMs. Most high-burden schools missed by the CRF were in central and northern regions, primarily around Lake Kivu and along the northern border with Uganda (Karongi, Rutsiro, Burera and Gicumbi; see Additional file 2: Figure S7 for locations of districts). In addition, three schools in Rwanda’s south (Nyamagabe and Nyaruguru) were high-burden schools that were missed by the CRF. In contrast, both models accurately identified low-burden schools. Sixty-six schools had no observed co-infections (SCR = 0); none of these were incorrectly classified by either model.

### Parasite associations as indicators of shared infection risk

We identified important demographic and environmental correlates of infection risk. Males were more likely to be infected with *S. mansoni* than were females (odds ratio (OR) 95% CI: 1.09–1.67). Infection by hookworms and by *A. lumbricoides* were both less likely to occur in districts with wider accessibility to household electricity (effect size 95% CIs: 0.67–0.83 and 0.83–0.97, respectively; Table 2). However, these parasites showed contrasting elevational gradients, with hookworm infection more likely in lower elevations and *A. lumbricoides* infection more likely at higher elevations (effect size 95% CIs: 0.57–0.69 and 1.60–1.92, respectively; Table 2). In addition, *A. lumbricoides* infection was more likely in older children (effect size 95% CI: 1.02–1.08) and less likely in areas with high vegetation density (effect size 95% CI: 0.79–0.95; Table 2). Infection by *T. trichiura* was less likely in croplands and areas with high NDVI (effect size 95% CI: 0.69–0.82) but more likely in areas with higher soil moisture (effect size 95% CI: 1.0–1.23; Table 2).

**Table 2:** CRF regression coefficients for predictors of each parasite’s infection probability

Parasite	Predictor	β coefficient (95% CI)
*Ascaris lumbricoides*	*T. trichiura* occurrence	1.15 (1.07–1.25)
	Elevation	0.57 (0.47–0.65)
	Proportion of human settlements classified as rural × *T. trichiura* occurrence	− 0.15 (− 0.29– − 0.03)
	NDVI	− 0.14 (− 0.23– − 0.05)
	Proportion of households with electricity	− 0.11 (− 0.19– − 0.03)
	Age	0.05 (0.02–0.08)
Hookworm spp.	*S. mansoni* occurrence	0.69 (0.24–1.06)
	*T. trichiura* occurrence	0.66 (0.49–0.82)
	Elevation	− 0.47 (− 0.56– − 0.37)
	Proportion of households with electricity	− 0.27 (− 0.39– − 0.19)
	Proportion of households with electricity × *T. trichiura* occurrence	− 0.22 (− 0.41– − 0.06)
*Schistosoma mansoni*	Hookworm occurrence	0.69 (0.24–1.06)
	*T. trichiura* occurrence	0.42 (0.05–0.72)
	Male	0.27 (0.09–0.51)
*Trichuris trichiura*	*A. lumbricoides* occurrence	1.15 (1.07–1.25)
	Hookworm occurrence	0.66 (0.49–0.82)
	NDVI	− 0.43 (− 0.53– − 0.30)
	*S. mansoni* occurrence	0.42 (0.05–0.72)
	Proportion of landcover classified as cropland	− 0.28 (− 0.37– − 0.20)
	Proportion of households with electricity × Hookworm spp. occurrence	− 0.22 (− 0.41– − 0.06)
	Surface soil moisture	0.15 (0.06–0.21)
	Proportion of human settlements classified as rural × *A. lumbricoides* occurrence	− 0.15 (− 0.29– − 0.03)

*Notes*: Only predictors whose 95% credible intervals (CIs) did not include zero are shown. Interaction effect between a co-occurring parasite and an environmental/demographic covariate is indicated by “×”

*Abbreviation*: NDVI, normalized difference vegetation index

Even after capturing effects of demographic and environmental covariates, associations with other parasites were strong predictors of risk (Table 2). For example, infection with *T. trichiura* was positively associated with the infection probabilities for each of the other parasites (Table 2). Co-infection between *A. lumbricoides* and *T. trichiura* was common, occurring in 1562 children (17.78%; Additional file 2: Figures S1, S2). Infection with one of these parasites led to a threefold increase in risk that a child would carry the other (OR: 2.92–3.49). ORs were 1.63–2.27 for the hookworm + *T. trichiura* association (found in 104 children) and 1.05–2.05 for the *S. mansoni + T. trichiura* association (found in 87 children). Even the two rarest parasites, hookworms and *S. mansoni*, were positively associated (Table 2; Additional file 2: Figure S2). Infection with one of these parasites led to more than a twofold increase in risk for the other (OR: 1.27–2.87).

A key finding was that some parasite co-infection prevalences showed marked variation across environmental or demographic gradients. For example, the most abundant co-infection, between *A. lumbricoides* and *T. trichiura*, was less likely to occur in areas encompassed by rural or undeveloped settlements than in urban/suburban areas (effect size 95% CI: 0.75–0.97; Table 2). In addition, co-infection between hookworms and *T. trichiura* were less likely to occur in districts with wider accessibility to electricity (effect size 95% CI: 0.66–0.94; Table 2).

### Delineating geographical areas with at-risk populations

**Fig. 1:**
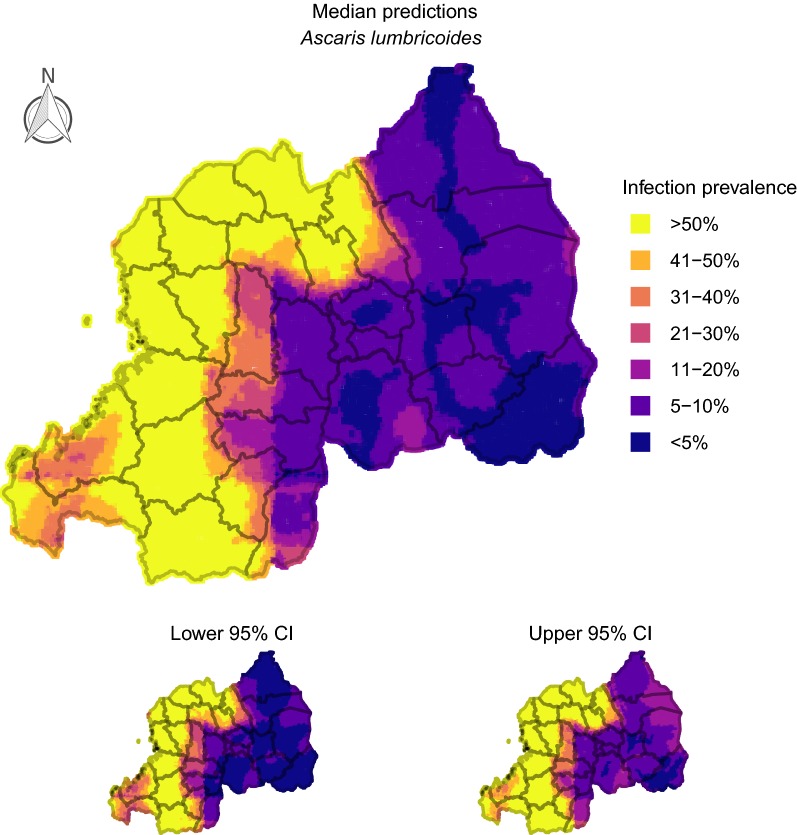
Predicted prevalence of *Ascaris lumbricoides* in Rwandan schoolchildren. 100 iterations of a spatial conditional random fields model were used to generate 95% credible prediction intervals (CIs). This figure was produced in R 3.5 using a shapefile representing Rwanda’s current administrative units [obtained from the data warehouse DIVA GIS (https://www.diva-gis.org/Data)]

**Fig. 2:**
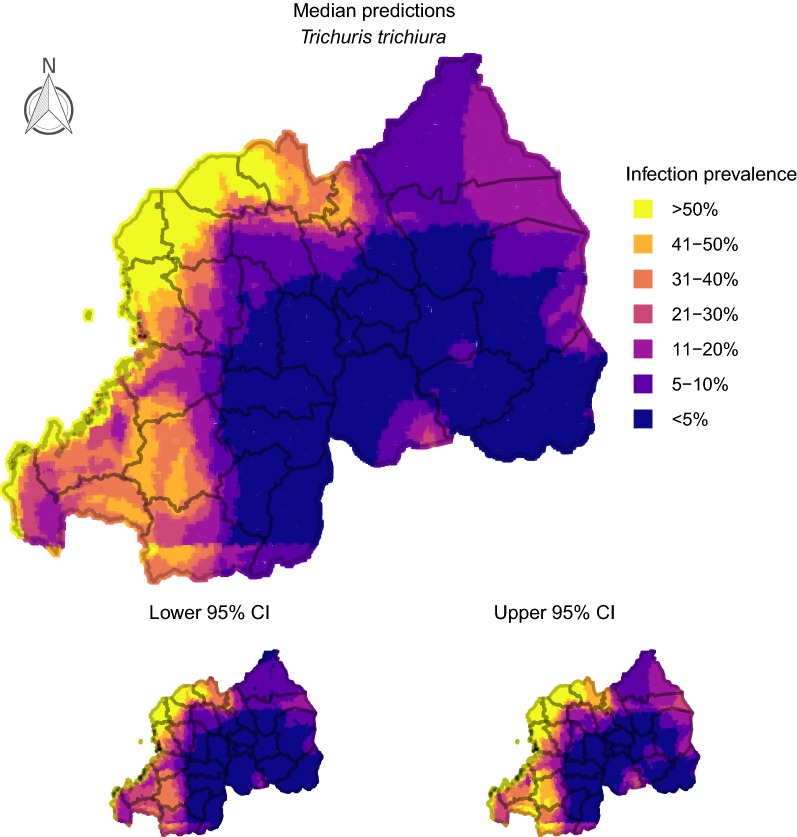
Predicted prevalence of *Trichuris trichiura* in Rwandan schoolchildren. 100 iterations of a spatial conditional random fields model were used to generate 95% credible prediction intervals (CIs). This figure was produced in R 3.5 using a shapefile representing Rwanda’s current administrative units [obtained from the data warehouse DIVA GIS (https://www.diva-gis.org/Data)]

**Fig. 3:**
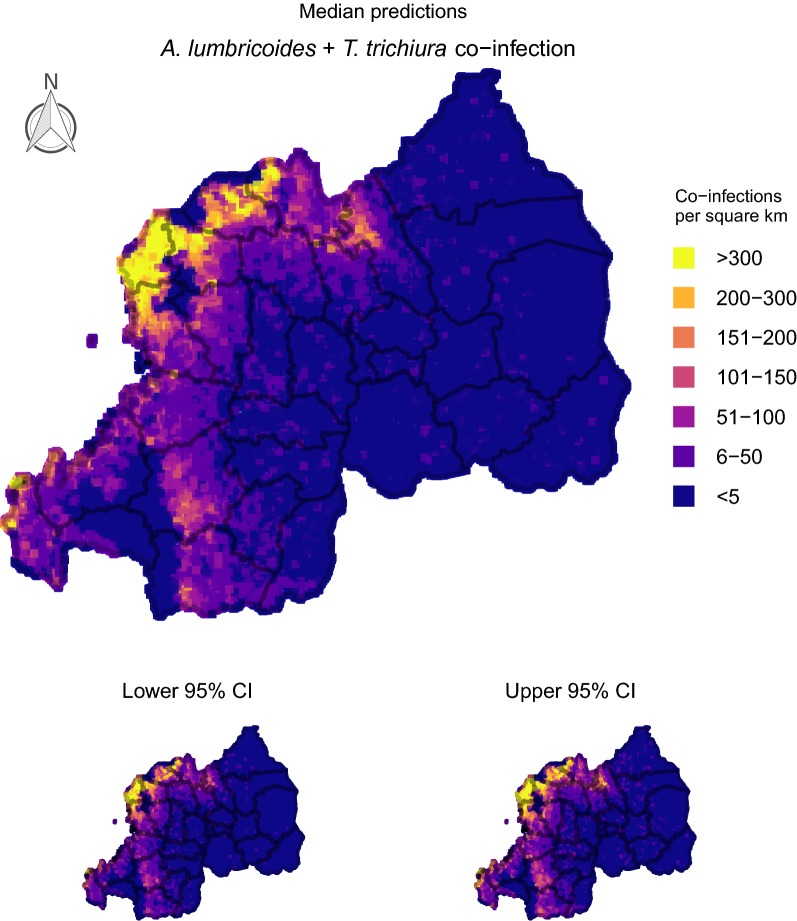
Predicted number of Rwandan children (aged 8–18 years-old) harbouring *A. lumbricoides*/*T. trichiura* co-infections. 100 iterations of a spatial conditional random fields model were used to generate 95% credible prediction intervals (CIs). This figure was produced in R 3.5 using a shapefile representing Rwanda’s current administrative units (obtained from the data warehouse DIVA GIS (https://www.diva-gis.org/Data))

Conditional prevalence predictions for the two most common helminths (*A. lumbricoides* and *T. trichiura*) delineated large spatial clusters that were generally robust to prediction uncertainty and would be considered ‘high risk’ according to WHO guidelines (areas with at least 50% infection prevalence; [65]). For *A. lumbricoides*, this at-risk cluster encompassed most of the country’s western districts, as well as many areas in the central-north and central-south (ranging from Nyaruguru in the south around the border to Gicumbi in the north; Fig. 1). High-risk clusters for *T. trichiura* were less extensive but occurred across similar geographical areas, with districts such as Rutsiro, Rubavu, Nyabihu and Musanze particularly impacted (Fig. 2). In fact, our models estimated that the burden of *A. lumbricoides* + *T. trichiura* co-infections strongly reflected the risk map for *T. trichiura*, with up to 300 or more children (aged 8–18 years) per km^2^ estimated to be co-infected in some western/northwestern districts (Fig. 3). In contrast, predicted prevalence for hookworms and *S. mansoni* was less than 20% across the country (Additional file 2: Figures S8, S9), corresponding to recommendations of two treatments per child during primary school years [65]. Hookworms were the only parasites that showed highest risk in eastern areas, but these regions did not reach > 20% even when using highest credible estimates (Additional file 2: Figure S8).

### Exploring parasite associations: faecal egg counts correlate with infection risk

Higher values for *A. lumbricoides* epg were associated with an increase in the probability that a child would also be infected with *T. trichiura* (an increase of 4280 *A. lumbricoides* epg correlated with a 11.54% relative increase in *T. trichiura* infection risk). The effect of *T. trichiura* epg on *A. lumbricoides* infection probability was also positive, though this effect was weaker (an increase of 470 *T. trichiura* epg correlated with a 4.87% relative increase in *A. lumbricoides* infection risk). Other notable correlations were a relative decrease of 3.25% in *T. trichiura* infection risk with every 50 additional *A. duodenale* epg and a relative increase of 3.25% in *A. lumbricoides*/*T. trichiura* co-infection probability with every 20 additional *S. mansoni* epg.

## Discussion

Infection by more than one helminth parasite is a major problem across much of the world’s tropical and sub-tropical regions [11, 12, 14, 22, 23]. Monitoring studies screen for multiple parasites, yet models generally ignore this multivariate data when designing treatment guidelines. We developed a model-based approach for incorporating co-infection data to carry out predictive risk modelling. Helminth co-infections are common in Rwandan schoolchildren, and we demonstrated how CRFs outperform single-parasite models by using this information to produce prediction maps. We argue that our approach is especially useful when information on individual-level upstream risk factors is missing, and that such pipelines should be continuously updated to refine estimates and critique the effectiveness of treatment programmes [39, 41, 42].

Uncovering demographic and environmental correlates of infection is a central goal of disease modelling. Despite the public health threats that helminth parasites constitute in Rwanda [66], knowledge of local risk factors and spatial variation in prevalence is poor [8, 9]. The availability of remote sensing databases has expanded capacity to address these drawbacks by predicting infectious disease distributions [39, 43], and these data layers are commonly coupled with surveys of local demographics, sanitation practices and access to healthcare [15, 16, 67]. Our findings reinforced earlier studies regarding the factors that influence the distributions of these important parasites. For example, reliable access to electricity is expected to reflect improved sanitation practices and possibly a reduction in environmental contamination by infective parasite stages [15]. Our findings supported this hypothesis by showing that increased household electricity access correlated with decreased risk of infection for both hookworms and *A. lumbricoides*. Male children were more likely to be infected with *S. mansoni*, a result that was also found in a study comparing different diagnostic methods for detecting this parasite in Rwandan children [68]. Whether this reflects increased risky behaviour by male children, or perhaps different cultural expectations regarding contributions to household farming, requires further investigation. In addition, we found that *T. trichiura* infection was more likely in areas with higher soil moisture, reflecting that the infectious stages of these parasites thrive in tropical and subtropical areas with moist soil conditions [69]. We also found that *A. lumbricoides* infection was more likely in high elevation areas, supporting similar findings from studies in nearby East African countries [70] and reflecting this parasite’s common infection occurrence in high-elevation areas around Lake Kivu in the country’s Northwest. An unexpected finding was the lower infection risk for the two most common parasites (*T. trichiura* and *A. lumbricoides*) in areas with higher post-rainy season vegetation density (NDVI). Large at-risk clusters for both of these parasites encompassed most of the country’s western districts around Lake Kivu, which are high-elevation areas characterised by low precipitation. Our results could reflect previous evidence that low NDVI values are indicative of areas with increased survival and transmission rates for *T. trichiura* and *A. lumbricoides* [10], or perhaps could demonstrate how parasite-parasite associations at the individual level can capture large proportions of variance in infection probability and make it more challenging to detect comparatively weak environmental associations. Further adaptation of our methods to other host-parasite contexts will be helpful for understanding these potential trade-offs.

Yet while we identified important environmental/demographic predictors of helminth infection, the stronger performance of the CRF compared to single-parasite models suggests that information on other parasites can increase accuracy of risk estimates even when remote sensing data are available. The CRF outperformed single-parasite GBM models for classifying schools into treatment categories and for predicting levels of co-infection, without sacrificing performance of predictions for each parasite separately. Moreover, parasite effects were more strongly predictive of individual infection risk than were environmental or demographic variables for each of the four studied parasite species. Several studies note that soil-transmitted and water-related helminths share common transmission pathways, exposure routes and overlapping risk factors [2, 41, 71, 72]. Our study extends this knowledge by demonstrating that, in the absence of more robust indicators of upstream exposure, multi-parasite data can act as a reliable proxy for data-poor individual-level risk factors such as water, sanitation and hygiene (WaSH) access, nutrition, defecation practices, poverty indices or occupational variation. Risk-mapping studies have not yet recognised this rich information, instead relying on remote-sensing data for which concerns have been raised over temporal mismatch, incomplete coverage or areal unit problems [73, 74].

In addition to the improved accuracy our model achieved when delineating schools into treatment categories, a primary advantage of the CRF ensemble over single-parasite models was its superior ability to predict *A. lumbricoides* + *T. trichiura* co-infections. This is an attractive property when considering health risks of co-infections and that co-infection prevalence could serve as useful indicators of disadvantage or environmental contamination. We identified robust multi-parasite associations that were not predicted to vary much across environmental gradients, suggesting these associations represent true biological phenomena that can be used to gain deeper insights into (i) which pathophysiological factors underpin multiple parasite infections or (ii) the ecology of multiparasitism. Our evidence that presence of some parasites correlated with variation in epg for co-occurring parasites could support the latter. Parasite co-infections can have multiplicative impacts on clinical outcomes, and previous evidence has suggested that parasite ‘community’ compositions can alter fertilities or shedding rates of competing parasites [72]. Rwandan children infected with *A. lumbricoides* were more likely to carry *T. trichiura*, which could be evidence of synchronous shedding driven by a synergistic within-host interaction [75]. However, determining whether our findings represent a true parasite interaction, as opposed to reflecting shared upstream risk factors but no biological interaction among parasite species *per se*, requires further study.

Rwanda has a population of over 10.7 million and an area of 26,338 km^2^, making it one of Africa’s most densely populated countries [67]. Determining how to efficiently reduce parasite infections in this agricultural region is a priority, as helminth infection is a common cause of anaemia and subsequent hospitalization [67]. Current WHO treatment guidelines state that decisions around how often to deworm children should rely on estimates of infection prevalence within school catchments [65]. Under these guidelines, our results suggest decisions around when and where to treat for STH infections in Rwanda will be dominated by classifying schools using estimates of *A. lumbricoides* and *T. trichiura* prevalence. These common parasites had observed prevalence of 37.54 and 23.16%, suggesting STH parasites are persisting despite the delivery of 18 million doses of anthelmintics to over 4 million Rwandans following a 2008 survey [8]. However, infection risks were not spatially homogeneous. Districts such as Rubavu, Rutsiro and Musanze, along the country’s western and north-western borders, were predicted to harbour the most *A. lumbricoides* + *T. trichiura* co-infections, affecting up to 300 or more children per km^2^. Moreover, prevalence of *A. lumbricoides* and *T. trichiura* single infections was predicted to be above 50% in most of these areas, placing schools in those districts within the ‘high risk’ WHO category requiring twice-yearly treatment [65].

In contrast to *A. lumbricoides* and *T. trichiura*, hookworm and schistosome (*S. mansoni*) infections were rare. In fact, *S. mansoni* was so rare that its infection probability was relatively insensitive to our tested covariates, apart from the sex of the child. Considering that broad-spectrum, multi-parasite benzimidazole-based anthelmintics are the primary drugs used to reduce STH burdens [76], our findings suggest that hookworm estimates are unlikely to make substantial contributions to management decisions in Rwanda. While we did identify areas with moderate risk of hookworm that did not overlap with *A. lumbricoides* and *T. trichiura* high-risk zones, these areas did not reach the ‘high risk’ threshold of 50% [65]. Our *S. mansoni* maps likewise did not delineate high-risk areas. Guidelines for reducing schistosomiasis are similar to STH, albeit with different thresholds [65, 77]. In our case, predictions suggest that schools in Rwanda are ‘low risk’ for *S. mansoni*, with treatment occurring once on entry to primary school and again on exit.

The proliferation of network methods in epidemiology has led to exponential increases in the discovery of associations between co-occurring organisms [78, 79]. Our approach integrates multiple data sources to disentangle biotic and environmental effects on infection risk. This represents a step forward for disease modelling for two reasons. First, the ‘big data’ era has seen an explosion in the availability of complex, multi-structured databases. Developing likelihood-based pipelines to analyse these datasets is an emerging field that has only begun to be explored by epidemiologists [80]. The models applied here can process datasets with hundreds of parasites and substantially more covariates, allowing for greater efficiency during crucial stages of model exploration than can be achieved with competing approaches. Secondly, graphical network models, particularly for binary data, have been refined across a diversity of areas ranging from gene association studies to network relationships among language passages [35]. Because they are used in many disciplines and their properties are understood, graphical network models represent an ideal area that can see exciting developments in disease mapping applications.

A number of aspects of our analysis could be improved in future work. The KK method presents well-known challenges associated with helminth detection [81]. Hookworm eggs may not survive long periods following collection, and detection using KK screening is limited to people harbouring infections with egg-producing female worms or perhaps people harbouring high burdens [82]. Multiple diagnostics may be needed to rectify these issues. The circulating cathodic antigen test, detected in urine, is more sensitive for detecting *Schistosoma* parasites, and can lead to dramatically different estimates of prevalence [81]. In addition, open-access remote-sensed environmental variables such as those we used in our models can contain particular uncertainties that are commonly ignored when producing high-resolution raster maps [10, 43]. Detection of environmental risk factors may be hampered by spatial or temporal variation in sanitation procedures, which we were unable to account for in our study. While surveys of schoolchildren are the primary method for analysing helminth infections, surveys that include adults would provide additional information that could improve insights into population-level risk factors [76]. Exploration of outputs at key points along our model chain gives insights into limitations that can be tackled with future work. Our CRF achieved good fit to the observed data and correctly predicted up to 80% of positive infections, suggesting our risk maps can be highly useful for managers. However, a poorer fit from the CRF would not necessarily increase the uncertainty envelopes in our risk-maps, as our framework does not fully propagate uncertainties from the CRF through to the posterior simulation. Adapting particle filter or Kalman filter routines that are commonly used in forecasting to propagate uncertainty [83, 84] may be a viable option that is worth exploring in future efforts to improve our approach.

## Conclusions

Monitoring studies are essential for understanding how parasites are distributed across the landscape and for detecting factors that can be reliably used to predict infection risk. These surveys routinely screen individuals for the presence of multiple parasites, many of which share important facets of their epidemiological cycles, yet statistical models generally ignore this rich multivariate data when assessing risk factors and designing treatment guidelines. Our CRF ensemble approach showcases how multivariate computational approaches can improve predictions of infection and co-infection prevalence by leveraging this type of data in efficient algorithms. Approaches such as ours can be instrumental in the global effort to reduce and eventually eliminate neglected helminth infections in the world’s developing countries.

## Supplementary information

**Additional file 1.** R Code and raw infection/covariate data needed to replicate CRF and GBM analyses.

**Additional file 2: Figure S1.** Observed infection prevalence for the four studied helminth parasites across 177 schools in Rwanda. **Figure S2.** Parasite co-infections and conditional correlation coefficients. **Figure S3.** Out-of-sample classification accuracies of the multi-parasite conditional random fields (CRF) and single-parasite gradient boosted machine (GBM) models. **Figure S4.** Area under the curve of the receiver operating characteristics (AUCs) for school-wide infection prevalence predicted by the conditional random fields (CRF) and single-parasite gradient boosted machine (GBM) models. **Figure S5.** Sensitivities of the conditional random fields (CRF) and single-parasite gradient boosted machine (GBM) models for predicting individual-level co-infections between *A. lumbricoides* and *T. trichiura*. **Figure S6.** Pearsonʼs correlations between each school’s observed standardised *A. lumbricoides* + *T. trichiura* co-infection ratio (SCR) and predicted SCRs from the conditional random fields (CRF) and single-parasite gradient boosted machine (GBM) models. **Figure S7.** Map of Rwanda’s provencial districts. **Figure S8.** Predicted infection prevalence of hookworm in Rwandan schoolchildren. **Figure S9.** Predicted infection prevalence of *Schistosoma mansoni* in Rwandan schoolchildren.

## Data Availability

Data supporting the conclusions of this article are included within the article and its additional files. All infection data and R code to replicate analyses is included in Additional file 1.

## Abbreviations

AUC: area under the curve of the receiver operating characteristic
CRF: conditional random field
GBM: gradient boosted machine
LST: land surface temperature
NDVI: normalized difference vegetation index
NDWI: normalized difference water index
SCR: standardised co-infection ratio

## References

[1] Ortu G, Assoum M, Wittmann U, Knowles S, Clements M, Ndayishimiye O (2016). The impact of an 8-year mass drug administration programme on prevalence, intensity and co-infections of soil-transmitted helminthiases in Burundi. Parasit Vectors..

[2] Kightlinger LK, Seed JR, Kightlinger MB (1995). The epidemiology of *Ascaris lumbricoides*, *Trichuris trichiura*, and hookworm in children in the Ranomafana Rainforest, Madagascar. J Parasitol..

[3] Owada K, Nielsen M, Lau CL, Clements AC, Yakob L, Soares Magalhães RJ (2017). Measuring the effect of soil-transmitted helminth infections on cognitive function in children: systematic review and critical appraisal of evidence. Adv Parasitol..

[4] Lardans V, Dissous C (1998). Snail control strategies for reduction of schistosomiasis transmission. Parasitol Today..

[5] Grimes JE, Croll D, Harrison WE, Utzinger J, Freeman MC, Templeton MR (2014). The relationship between water, sanitation and schistosomiasis: a systematic review and meta-analysis. PLoS Negl Trop Dis..

[6] Pullan RL, Smith JL, Jasrasaria R, Brooker SJ (2014). Global numbers of infection and disease burden of soil transmitted helminth infections in 2010. Parasit Vectors..

[7] van der Werf MJ, de Vlas SJ, Brooker S, Looman CW, Nagelkerke NJ, Habbema JDF (2003). Quantification of clinical morbidity associated with schistosome infection in sub-Saharan Africa. Acta Trop..

[8] Rujeni N, Morona D, Ruberanziza E, Mazigo HD (2017). Schistosomiasis and soil-transmitted helminthiasis in Rwanda: an update on their epidemiology and control. Infect Dis Poverty..

[9] Staudacher O, Heimer J, Steiner F, Kayonga Y, Havugimana JM, Ignatius R (2014). Soil-transmitted helminths in southern highland Rwanda: associated factors and effectiveness of school-based preventive chemotherapy. Trop Med Int Health..

[10] Brooker S, Clements AC, Bundy DA (2006). Global epidemiology, ecology and control of soil-transmitted helminth infections. Adv Parasitol..

[11] Soares Magalhães RJ, Biritwum N-K, Gyapong JO, Brooker S, Zhang Y, Blair L (2011). Mapping helminth co-Infection and co-intensity: geostatistical prediction in Ghana. PLoS Negl Trop Dis..

[12] Raso G, Vounatsou P, Singer BH, Eliézer K, Tanner M, Utzinger J (2006). An integrated approach for risk profiling and spatial prediction of *Schistosoma mansoni*-hookworm coinfection. Proc Natl Acad Sci USA.

[13] Phiri BBW, Ngwira B, Kazembe LN (2016). Analysing risk factors of co-occurrence of schistosomiasis haematobium and hookworm using bivariate regression models: case study of Chikwawa, Malawi. Parasit Epidemiol Control..

[14] WHO. WHO Expert Committee on the Control of Schistosomiasis: prevention and control of schistosomiasis and soil-transmitted helminthiasis: report of a WHO expert committee. Geneva: World Health Organization; 2002. https://apps.who.int/iris/handle/10665/42588. Accessed 7 June 2019. 12592987

[15] Campbell SJ, Savage GB, Gray DJ, Atkinson JAM, Soares Magalhães RJ, Nery SV (2014). Water, Sanitation, and Hygiene (WASH): a critical component for sustainable soil-transmitted helminth and schistosomiasis control. PLoS Negl Trop Dis..

[16] Hotez P (2009). Mass drug administration and integrated control for the world’s high-prevalence neglected yropical diseases. Clin Pharmacol Ther..

[17] Soares Magalhães RJ, Clements ACA, Patil AP, Gething PW, Brooker S (2011). The applications of model-based geostatistics in helminth epidemiology and control. Adv Parasitol..

[18] Fenwick A, Jourdan P (2016). Schistosomiasis elimination by 2020 or 2030?. Int J Parasitol..

[19] World Bank: The World Bank in Rwanda. 2014 http://www.worldbank.org/en/country/rwanda/overview. Accessed 6 June 2019.

[20] Pullan RL, Kabatereine NB, Quinnell RJ, Brooker S (2010). Spatial and genetic epidemiology of hookworm in a rural community in Uganda. PLoS Negl Trop Dis..

[21] Clements AC, Firth S, Dembelé R, Garba A, Touré S, Sacko M (2009). Use of Bayesian geostatistical prediction to estimate local variations in *Schistosoma haematobium* infection in western Africa. Bull World Health Organ..

[22] Brooker S, Clements AC (2009). Spatial heterogeneity of parasite co-infection: determinants and geostatistical prediction at regional scales. Int J Parasitol..

[23] Owada K, Lau CL, Leonardo L, Clements ACA, Yakob L, Nielsen M (2018). Spatial distribution and populations at risk of *A. lumbricoides* and *T. trichiura* co-infections and infection intensity classes: an ecological study. Parasit Vectors..

[24] Bisanzio D, Mutuku F, Bustinduy AL, Mungai PL, Muchiri EM, King CH (2014). Cross-sectional study of the burden of vector-borne and soil-transmitted polyparasitism in rural communities of Coast Province, Kenya. PLoS Negl Trop Dis..

[25] Fleming FM, Brooker S, Geiger SM, Caldas IR, Correa-Oliveira R, Hotez PJ (2006). Synergistic associations between hookworm and other helminth species in a rural community in Brazil. Trop Med Int Health..

[26] Popovic GC, Warton DI, Thomson FJ, Hui FKC, Moles AT (2019). Untangling direct species associations from indirect mediator species effects with graphical models. Methods Ecol Evol..

[27] Ovaskainen O, Hottola J, Siitonen J (2010). Modeling species co-occurrence by multivariate logistic regression generates new hypotheses on fungal interactions. Ecology..

[28] Chesson P, Gebauer RLE, Schwinning S, Huntly N, Wiegand K, Ernest MSK (2004). Resource pulses, species interactions, and diversity maintenance in arid and semi-arid environments. Oecologia..

[29] Ovaskainen O, Tikhonov G, Norberg A, Guillaume Blanchet F, Duan L, Dunson D (2017). How to make more out of community data? A conceptual framework and its implementation as models and software. Ecol Lett..

[30] Golding N, Nunn MA, Purse BV (2015). Identifying biotic interactions which drive the spatial distribution of a mosquito community. Parasit Vectors..

[31] Clark NJ, Wells K, Dimitrov D, Clegg SM (2016). Co-infections and environmental conditions drive the distributions of blood parasites in wild birds. J Anim Ecol..

[32] Fountain-Jones NM, Packer C, Jacquot M, Blanchet FG, Terio K, Craft ME (2019). Endemic infection can shape exposure to novel pathogens: pathogen co-occurrence networks in the Serengeti lions. Ecol Lett..

[33] Clark NJ, Wells K, Lindberg O (2018). Unravelling changing interspecific interactions across environmental gradients using Markov random fields. Ecology..

[34] Kenduiywo B, Bargiel D, Soergel U (2016). Crop type mapping from a sequence of TerraSAR-X images with dynamic conditional random fields. ISPRS J Photogramm Remote Sens..

[35] Li B, Chun H, Zhao H (2012). Sparse estimation of conditional graphical models with application to gene networks. J Am Stat Assoc..

[36] Cheng J, Levina E, Wang P, Zhu J (2014). A sparse Ising model with covariates. Biometrics..

[37] Ruberanziza E, Owada K, Clark NJ, Umulisa I, Ortu G, Lancaster W (2019). Mapping soil-transmitted helminth parasite infection in Rwanda: estimating endemicity and identifying at-risk populations. Trop Med Infect Dis..

[38] WHO. Basic laboratory methods in medical parasitology. Geneva: World Health Organization; 1991. http://whqlibdoc.who.int/publications/9241544104_(part1).pdf?ua=1. Accessed 12 Apr 2019.

[39] Brooker S, Michael E (2000). The potential of geographical information systems and remote sensing in the epidemiology and control of human helminth infections. Adv Parasitol..

[40] Traub RJ (2013). *Ancylostoma ceylanicum*, a re-emerging but neglected parasitic zoonosis. Int J Parasitol..

[41] Pullan RL, Gething PW, Smith JL, Mwandawiro CS, Sturrock HJ, Gitonga CW (2011). Spatial modelling of soil-transmitted helminth infections in Kenya: a disease control planning tool. PLoS Negl Trop Dis..

[42] Truscott J, Turner H, Farrell S, Anderson R (2016). Soil-transmitted helminths: Mathematical models of transmission, the impact of mass drug administration and transmission elimination criteria. Adv Parasitol..

[43] Hay S, Tatem A, Graham A, Goetz S, Rogers D (2006). Global environmental data for mapping infectious disease distribution. Adv Parasitol..

[44] Brooker S, Alexander N, Geiger S, Moyeed RA, Stander J, Fleming F (2006). Contrasting patterns in the small-scale heterogeneity of human helminth infections in urban and rural environments in Brazil. Int J Parasitol..

[45] Appleton C (1978). Review of literature on abiotic factors influencing the distribution and life cycles of bilharziasis intermediate host snails. Malacol Rev..

[46] Gizaw Z, Adane T, Azanaw J, Addisu A, Haile D (2018). Childhood intestinal parasitic infection and sanitation predictors in rural Dembiya, northwest Ethiopia. Environ Health Prev Med..

[47] Anderson R, Truscott J, Hollingsworth TD (2014). The coverage and frequency of mass drug administration required to eliminate persistent transmission of soil-transmitted helminths. Philos Trans R Soc Lond B Biol Sci..

[48] Malaria and other parasitic diseases division of the Rwanda Biomedical Center Ministry of Health (Rwanda) and ICF. Rwanda malaria indicator survey (RMIS) 2017. Kigali, Rwanda, and Rockville, Maryland, USA: MOPDD and ICF. https://dhsprogram.com/pubs/pdf/MIS30/MIS30.pdf. Accessed 17 June 2019.

[49] Koller D, Friedman N (2009). Probabilistic graphical models: principles and techniques.

[50] Lindberg O. Markov random fields in cancer mutation dependencies. M.Sc. Thesis, University of Turku, Finland; 2016.

[51] Wainwright M, Ravikumar P, Lafferty J (2006). High-dimensional graphical model selection using l1-regularized logistic regression. NIPS..

[52] Friedman J, Hastie T, Tibshirani R (2010). Regularization paths for generalized linear models *via* coordinate descent. J Stat Softw..

[53] Dormann CF, Elith J, Bacher S, Buchmann C, Carl G, Carré G (2013). Collinearity: a review of methods to deal with it and a simulation study evaluating their performance. Ecography..

[54] Peel AJ, Wells K, Giles J, Boyd V, Burroughs A, Edson D (2019). Synchronous shedding of multiple bat paramyxoviruses coincides with peak periods of Hendra virus spillover. Emerg Microbes Infect..

[55] Fountain-Jones NM, Clark NJ, Kinsley AC, Carstensen M, Forester J, Johnson TJ (2019). Microbial associations and spatial proximity predict North American moose (*Alces alces*) gastrointestinal community composition. J Anim Ecol..

[56] Gruber S, Logan RW, Jarrín I, Monge S, Hernán MA (2015). Ensemble learning of inverse probability weights for marginal structural modeling in large observational datasets. Stat Med..

[57] Li F, Xu L, Siva P, Wong A, Clausi DA (2015). Hyperspectral image classification with limited labeled training samples using enhanced ensemble learning and conditional random fields. IEEE J STARS..

[58] Galar M, Fernandez A, Barrenechea E, Bustince H, Herrera F (2012). A review on ensembles for the class imbalance problem: bagging-, boosting-, and hybrid-based approaches. IEEE Trans Syst Man Cybern Syst..

[59] Miller PJ, Lubke GH, McArtor DB, Bergeman CS (2016). Finding structure in data using multivariate tree boosting. Psychol Methods..

[60] Elith J, Leathwick JR, Hastie T (2008). A working guide to boosted regression trees. J Anim Ecol..

[61] Clark NJ, Wells K, Lindberg O: MRFcov: Markov random fields with additional covariates. R package version 1.0. 2018. https://github.com/nicholasjclark/MRFcov.

[62] Brock PM, Fornace KM, Grigg MJ, Anstey NM, William T, Cox J (2019). Predictive analysis across spatial scales links zoonotic malaria to deforestation. Proc Roy Soc B Biol Sci..

[63] Fang LQ, Li XL, Liu K, Li YJ, Yao HW, Liang S (2013). Mapping spread and risk of avian influenza A (H7N9) in China. Sci Rep..

[64] National Institute of Statistics of Rwanda (NISR): fourth population and housing census, Rwanda, 2012. Ministry of Finance and Economic Planning. Kigali, Rwanda. http://www.statistics.gov.rw/survey-period/fourth-population-and-housing-census-2012. Accessed 24 July 2019.

[65] WHO. Helminth control in school-age children: a guide for managers of control programmes, 2nd edition. Geneva: World Health Organization; 2002. http://apps.who.int/iris/bitstream/10665/44671/1/9789241548267_eng.pdf?ua=1. Accessed 14 June 2019.

[66] Rwanda Human Resources for Health Program: strategic plan 2011–2016. Kigali. Ministry of Health of the Republic of Rwanda. 2011. http://www.brown.edu/academics/medical/bright/sites/brown.edu.academics.medical.bright/files/uploads/MOH%20Rwanda%20HRH%20Strategic%20Plan%202011%20-%202016.pdf. Accessed 14 June 2019.

[67] ICF International: Rwanda demographic and health survey 2014–15, National Institute of Statistics of Rwanda. In: The DHS Program. Rockville, Maryland, USA; 2016. https://dhsprogram.com/pubs/pdf/FR316/FR316.pdf. Accessed 11 May 2019.

[68] Clark NJ, Umulisa I, Ruberanziza E, Owada K, Colley DG, Ortu G (2019). Mapping *Schistosoma mansoni* endemicity in Rwanda: a critical assessment of geographical disparities arising from circulating cathodic antigen *versus* Kato-Katz diagnostics. PLoS Negl Trop Dis..

[69] Bethony J, Brooker S, Albonico M, Geiger SM, Loukas A, Diemert D (2006). Soil-transmitted helminth infections: ascariasis, trichuriasis, and hookworm. Lancet..

[70] Clements ACA, Deville MA, Ndayishimiye O, Brooker S, Fenwick A (2010). Spatial co-distribution of neglected tropical diseases in the East African Great Lakes region: revisiting the justification for integrated control. Trop Med Int Health..

[71] Traub RJ, Robertson ID, Irwin P, Mencke N, Andrew Thompson R (2004). The prevalence, intensities and risk factors associated with geohelminth infection in tea-growing communities of Assam, India. Trop Med Int Health..

[72] Pullan R, Brooker S (2008). The health impact of polyparasitism in humans: are we under-estimating the burden of parasitic diseases?. Parasitology..

[73] Navas ALA, Hamm NA, Magalhaes RJS, Stein A (2016). Mapping soil transmitted helminths and schistosomiasis under uncertainty: a systematic review and critical appraisal of evidence. PLoS Negl Trop Dis..

[74] Atkinson PM, Graham A (2006). Issues of scale and uncertainty in the global remote sensing of disease. Adv Parasitol..

[75] Ezeamama AE, McGarvey ST, Acosta LP, Zierler S, Manalo DL, Wu HW (2008). The synergistic effect of concomitant schistosomiasis, hookworm, and *Trichuris* infections on children’s anemia burden. PLoS Negl Trop Dis..

[76] Campbell S, Osei-Atweneboana M, Stothard R, Koukounari A, Cunningham L, Armoo S (2018). The COUNTDOWN study protocol for expansion of mass drug administration strategies against schistosomiasis and soil-transmitted helminthiasis in Ghana. Trop Med Infect Dis..

[77] WHO. Preventive chemotherapy in human helminthiasis. Geneva: World Health Organization; 2006. https://apps.who.int/iris/bitstream/handle/10665/43545/9241547103_eng.pdf;jsessionid=DCC18D3A05E2B6ED0F5E29ACADEDC253?sequence=1. Accessed 11 July 2019.

[78] Morand S, McIntyre KM, Baylis M (2014). Domesticated animals and human infectious diseases of zoonotic origins: domestication time matters. Infect Gen Evol..

[79] Wells K, Feldhaar H, O’Hara RB (2014). Population fluctuations affect inference in ecological networks of multi-species interactions. Oikos..

[80] Pfeiffer DU, Stevens KB (2015). Spatial and temporal epidemiological analysis in the Big Data era. Prev Vet Med..

[81] Kittur N, Castleman JD, Campbell CH, King CH, Colley DG (2016). Comparison of *Schistosoma mansoni* prevalence and intensity of infection, as determined by the circulating cathodic antigen urine assay or by the Kato-Katz fecal assay: a systematic review. Am J Trop Med Hyg..

[82] Foo KT, Blackstock AJ, Ochola EA, Matete DO, Mwinzi PN, Montgomery SP (2015). Evaluation of point-of-contact circulating cathodic antigen assays for the detection of *Schistosoma mansoni* infection in low-, moderate-, and high-prevalence schools in western Kenya. Am J Trop Med Hyg..

[83] Dietze MC (2017). Prediction in ecology: a first-principles framework. Ecol Appl..

[84] Massoud EC, Huisman J, Benincà E, Dietze MC, Bouten W, Vrugt JA (2018). Probing the limits of predictability: data assimilation of chaotic dynamics in complex food webs. Ecol Lett..

