# Supplementary material for: Parasite associations predict infection risk: incorporating co-infections in predictive models for neglected tropical diseases
Source: Parasit Vectors. 2020 Mar 16;13:138. doi: 10.1186/s13071-020-04016-2 (PMC7077138; doi:10.1186/s13071-020-04016-2)
Supplement: Supplementary file 2 — Additional file 2: Figure S1. Observed infection prevalence for the four studied helminth parasites across 177 schools in Rwanda. Figure S2. Parasite co-infections and conditional correlation coefficients. Figure S3. Out-of-sample classification accuracies of the multi-parasite conditional random fields (CRF) and single-parasite gradient boosted machine (GBM) models. Figure S4. Area under the curve of the receiver operating characteristics (AUCs) for school-wide infection prevalence predicted by the conditional random fields (CRF) and single-parasite gradient boosted machine (GBM) models. Figure S5. Sensitivities of the conditional random fields (CRF) and single-parasite gradient boosted machine (GBM) models for predicting individual-level co-infections between A. lumbricoides and T. trichiura. Figure S6. Pearsonʼs correlations between each school’s observed standardised A. lumbricoides + T. trichiura co-infection ratio (SCR) and predicted SCRs from the conditional random fields (CRF) and single-parasite gradient boosted machine (GBM) models. Figure S7. Map of Rwanda’s provencial districts. Figure S8. Predicted infection prevalence of hookworm in Rwandan schoolchildren. Figure S9. Predicted infection prevalence of Schistosoma mansoni in Rwandan schoolchildren. [file 13071_2020_4016_MOESM2_ESM.docx]

**Additional file 2: Additional Figures for**

**Parasite associations predict infection risk: incorporating co-infections in predictive models for neglected tropical diseases**

Nicholas J. Clark^1,2*^, Kei Owada^1,2,^ , Eugene Ruberanziza^3^, Giuseppina Ortu^4^, Irenee Umulisa^3^, Ursin Bayisenge^3^, Jean Bosco Mbonigaba^3^, Jean Bosco Mucaca^5^, Warren Lancaster^6^, Alan Fenwick^4^, Ricardo J. Soares Magalhães^1,2^ and Aimable Mbituyumuremyi^7^

^1^ UQ Spatial Epidemiology Laboratory, School of Veterinary Science, the University of Queensland, Gatton 4343, Queensland, Australia

^2^ Children Health and Environment Program, Child Health Research Centre, The University of Queensland, South Brisbane 4101, Queensland, Australia

^3^ Neglected Tropical Diseases and Other Parasitic Diseases Unit, Malaria and Other Parasitic Diseases Division, Rwanda Biomedical Center, Kigali, Rwanda

^4^ Schistosomiasis Control Initiative (SCI), Department of Infectious Diseases Epidemiology, Imperial College, London, United Kingdom

^5^ Microbiology Unit, National Reference Laboratory (NRL) Division, Rwanda Biomedical Center, Ministry of Health, Kigali, Rwanda

^6^ The END Fund, 2 Park Avenue, 18th Floor, New York, NY, 10016, USA

^7^ Malaria and Other Parasitic Diseases Division, Rwanda Biomedical Center, Ministry of Health, Kigali, Rwanda

^*^ Corresponding author:

Nicholas J. Clark

nicholas.j.clark1214@gmail.com


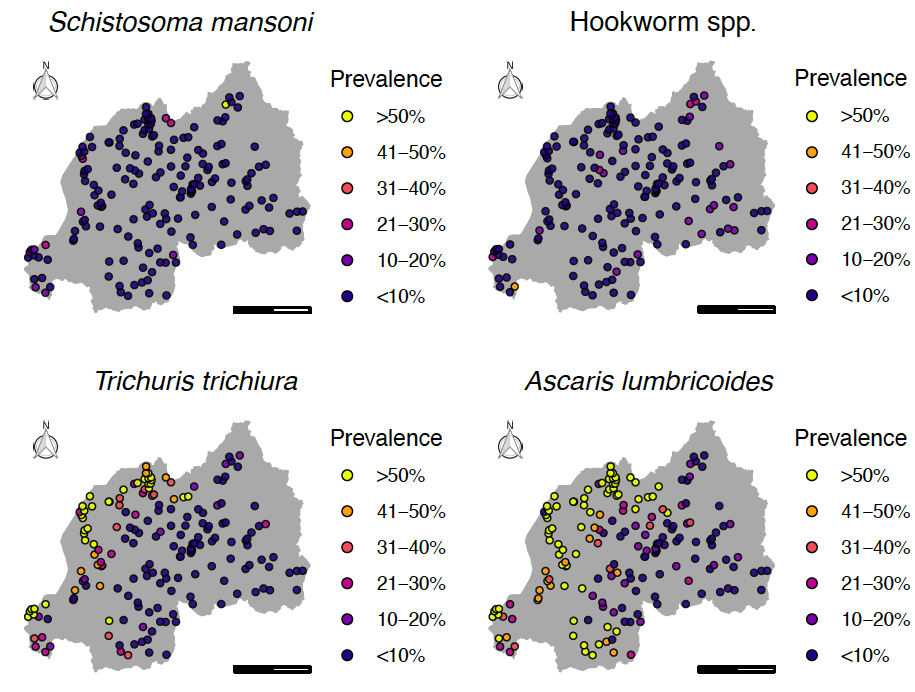


**Figure S1**: Observed infection prevalence for the four studied helminth parasites across 177 schools in Rwanda. The scale bar represents 60km. This figure was produced in R 3.5 using a shapefile representing Rwanda’s current administrative units (obtained from the data warehouse DIVA GIS ([www.diva-gis.org/Data)](http://www.diva-gis.org/Data))).


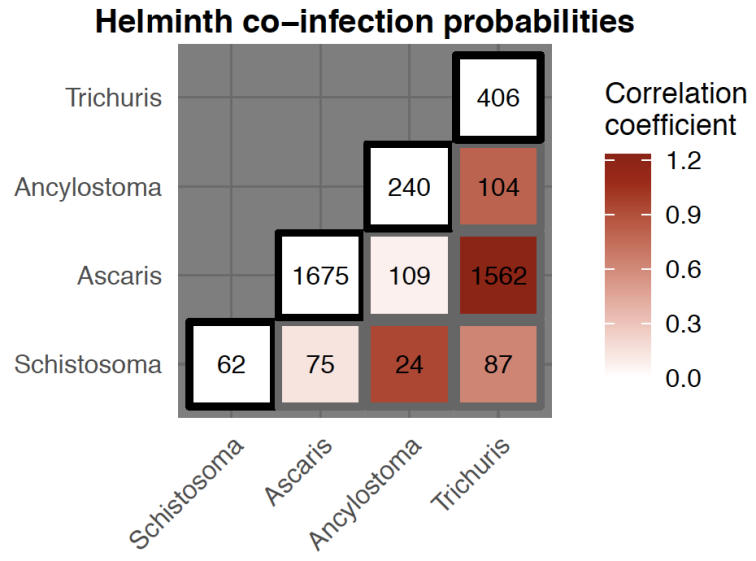


**Figure S2:** Conditional correlations in parasite co-infection probabilities. Off-diagonal values represent the total number of co-infections observed for each parasite pair from a sample of 8,786 children in Rwanda. Diagonals represent the number of single infections detected for each parasite. Some children were infected with more than two parasites. Coefficients were estimated from a spatially adjusted Conditional Random Fields model. Darker reds indicate that a parasite pair’s infection risks are more strongly associated after accounting for spatial processes and environmental variation. Note, correlations represent regression coefficients for a parasite’s log-odds and can therefore exceed 1.


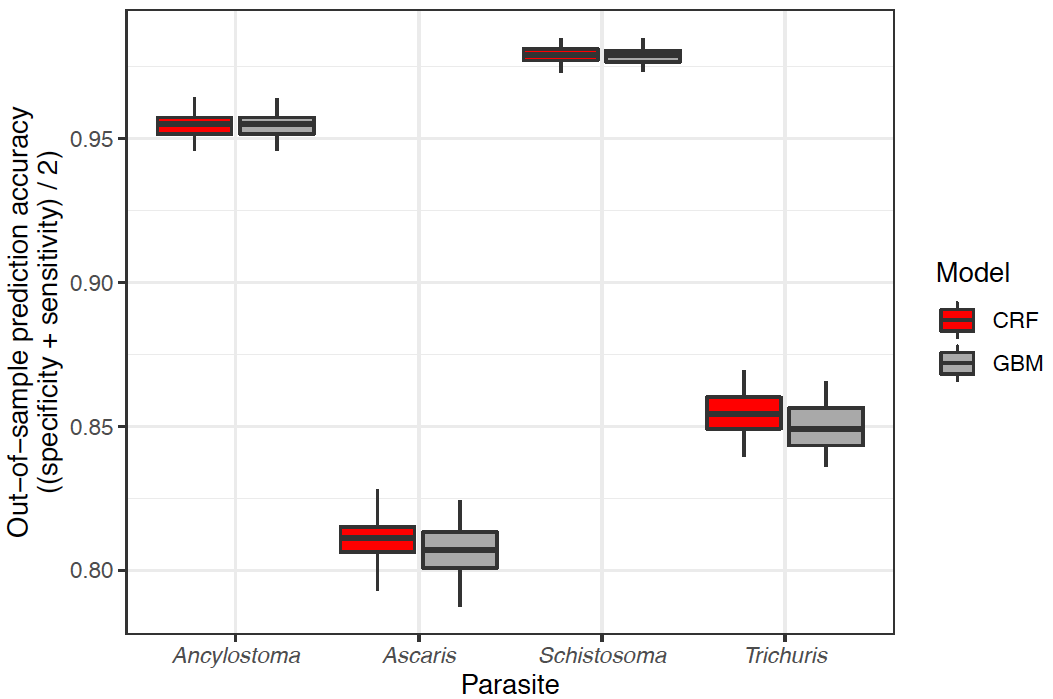


**Figure S3**: Out-of-sample classification accuracies of the multi-parasite Conditional Random Fields (CRF) and single-parasite gradient boosted machine (GBM) models. Boxes show medians (black lines), interquartile ranges (hinges) and range (whiskers).

**
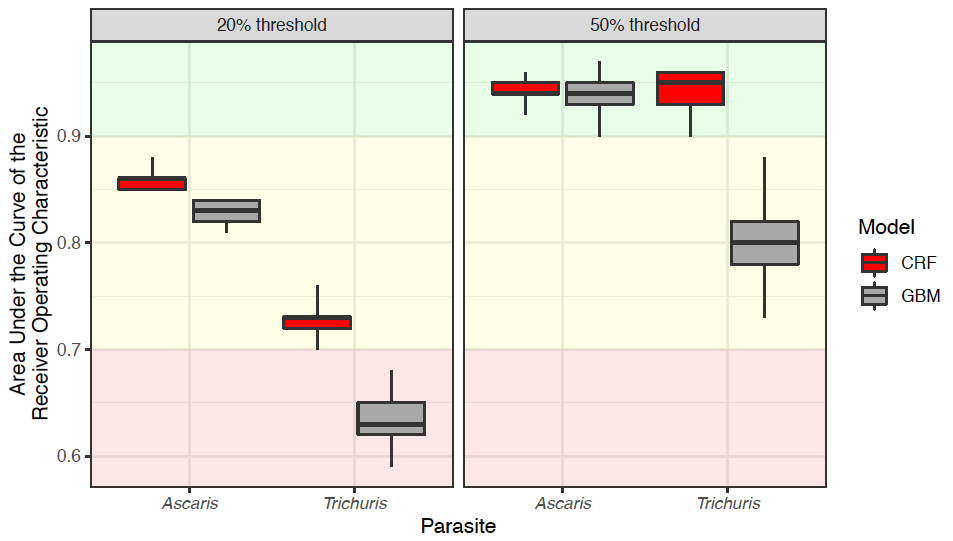
**

**Figure S4**: Area Under the Curve of the Receiver Operating Characteristics (AUCs) for school-wide infection prevalence predicted by the Conditional Random Fields (CRF) and single-parasite gradient boosted machine (GBM) models. Shaded zones represent AUCs indicative of good (green), reasonable (yellow) and poor (red) discriminatory power. Boxes show medians (black lines), interquartile ranges (hinges) and range (whiskers).


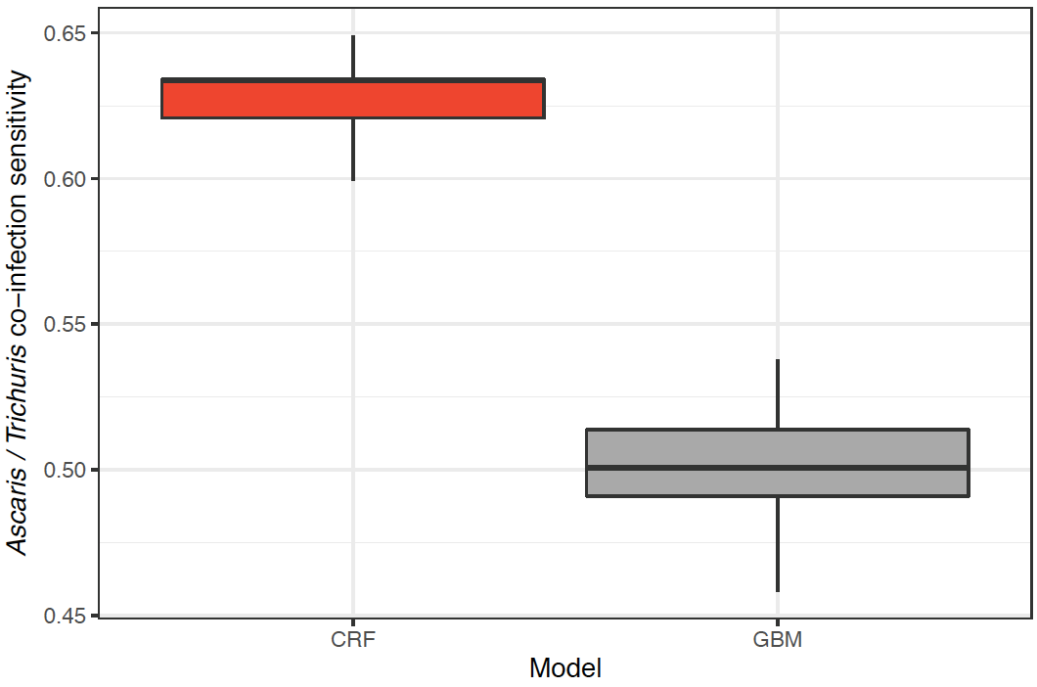


**Figure S5**: Sensitivities of the Conditional Random Fields (CRF) and single-parasite gradient boosted machine (GBM) models for predicting individual-level co-infections between *A. lumbricoides* and *T. trichiura*. Boxes show medians (black lines), interquartile ranges (hinges) and range (whiskers).


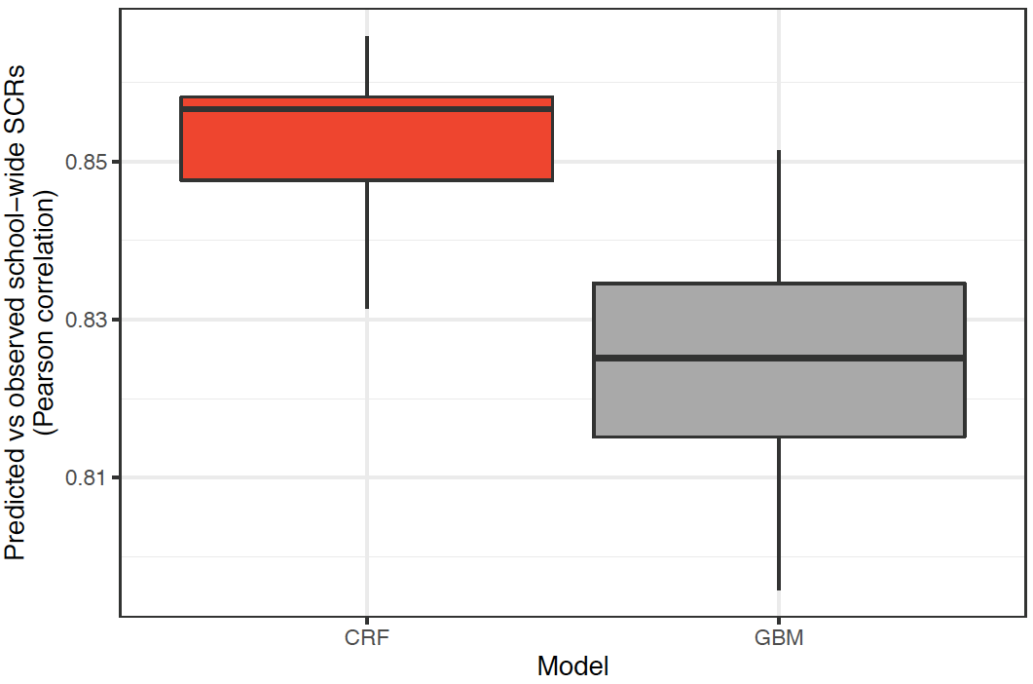


**Figure S6**: Pearson correlations between each school’s observed Standardised *A. lumbricoides* / *T. trichiura* Co-infection Ratio (SCR) and predicted SCRs from the Conditional Random Fields (CRF) and single-parasite gradient boosted machine (GBM) models. Boxes show medians (black lines), interquartile ranges (hinges) and range (whiskers).

**Figure S7**: Map of Rwanda’s provincial districts. This figure was produced in R version 3.5 using a shapefile representing Rwanda’s current administrative units (obtained from the geographic data warehouse DIVA GIS ([www.diva-gis.org/Data)](http://www.diva-gis.org/Data))).


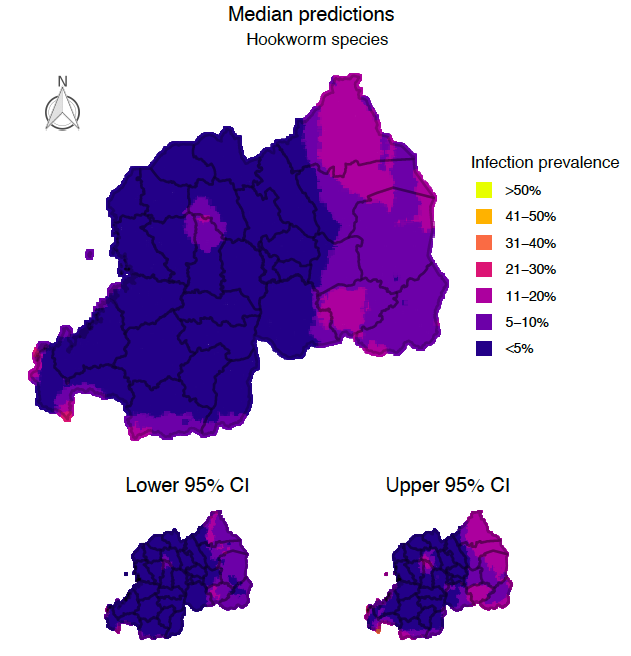


**Figure S8:** Predicted infection prevalence of hookworm in Rwandan schoolchildren. 100 iterations of a spatially adjusted Conditional Random Fields model were used to generate 95% credible prediction intervals (CIs). This figure was produced in R 3.5 using a shapefile representing Rwanda’s current administrative units (obtained from the data warehouse DIVA GIS ([www.diva-gis.org/Data)](http://www.diva-gis.org/Data))).


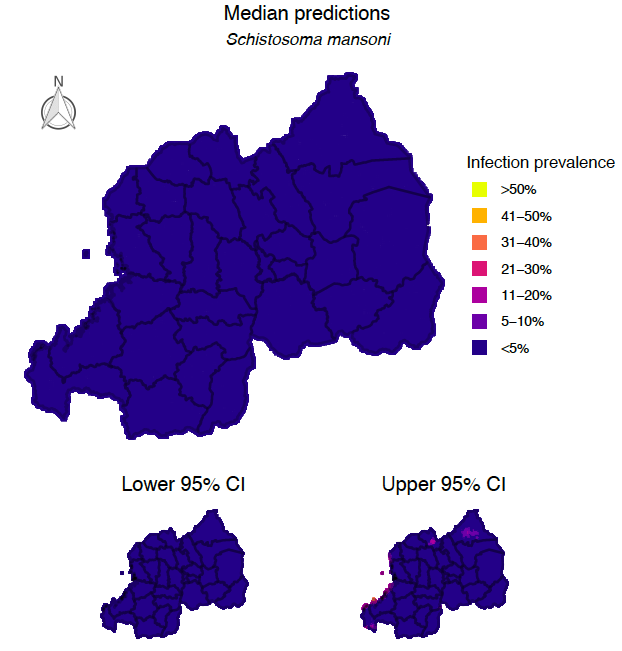


**Figure S9:** Predicted infection prevalence of *Schistosoma mansoni* in Rwandan schoolchildren. 100 iterations of a spatially adjusted Conditional Random Fields model were used to generate 95% credible prediction intervals (CIs). This figure was produced in R 3.5 using a shapefile representing Rwanda’s current administrative units (obtained from the data warehouse DIVA GIS ([www.diva-gis.org/Data)](http://www.diva-gis.org/Data))).
